# Mutant p53 and Twist1 Co-Expression Predicts Poor Prognosis and Is an Independent Prognostic Factor in Breast Cancer

**DOI:** 10.3389/fonc.2021.628814

**Published:** 2021-06-24

**Authors:** Yong-Qu Zhang, Fan Zhang, Yun-Zhu Zeng, Min Chen, Wen-He Huang, Jun-Dong Wu, Wei-Ling Chen, Wen-Liang Gao, Jing-Wen Bai, Rui-Qin Yang, Huan-Cheng Zeng, Xiao-Long Wei, Guo-Jun Zhang

**Affiliations:** ^1^ Department of Breast-Thyroid-Surgery and Cancer Research Center, Xiang’an Hospital of Xiamen University, Xiamen, China; ^2^ Clinical Central Research Core, School of Medicine, Xiang’an Hospital of Xiamen University, Xiamen, China; ^3^ Key Laboratory for Endocrine-Related Cancer Precision Medicine of Xiamen, Xiang’an Hospital of Xiamen University, Xiamen, China; ^4^ Cancer Research Center, School of Medicine, Xiamen University, Xiamen, China; ^5^ Guangdong Provincial Key Laboratory for Breast Cancer Diagnosis and Treatment, Cancer Hospital of Shantou University Medical College, Shantou, China; ^6^ Department of Pathology, Cancer Hospital of Shantou University Medical College, Shantou, China; ^7^ Department of Breast Center, Cancer Hospital of Shantou University Medical College, Shantou, China; ^8^ Department of Medical Oncology, Xiang’an Hospital of Xiamen University, Xiamen, China

**Keywords:** mutant p53, Twist1, breast cancer, independent prognostic factor, poor prognosis

## Abstract

**Purpose:**

The basic helix-loop-helix transcription factor (bHLH) transcription factor Twist1 plays a key role in embryonic development and tumorigenesis. p53 is a frequently mutated tumor suppressor in cancer. Both proteins play a key and significant role in breast cancer tumorigenesis. However, the regulatory mechanism and clinical significance of their co-expression in this disease remain unclear. The purpose of this study was to analyze the expression patterns of p53 and Twist1 and determine their association with patient prognosis in breast cancer. We also investigated whether their co-expression could be a potential marker for predicting patient prognosis in this disease.

**Methods:**

Twist1 and mutant p53 expression in 408 breast cancer patient samples were evaluated by immunohistochemistry. Kaplan-Meier Plotter was used to analyze the correlation between co-expression of Twist1 and wild-type or mutant p53 and prognosis for recurrence-free survival (RFS) and overall survival (OS). Univariate analysis, multivariate analysis, and nomograms were used to explore the independent prognostic factors in disease-free survival (DFS) and OS in this cohort.

**Results:**

Of the 408 patients enrolled, 237 (58%) had high mutant p53 expression. Two-hundred twenty patients (53.9%) stained positive for Twist1, and 188 cases were Twist1-negative. Furthermore, patients that co-expressed Twist1 and mutant p53 (T+P+) had significantly advanced-stage breast cancer [stage III, 61/89 T+P+ (68.5%) *vs.* 28/89 T-P- (31.5%); stage II, 63/104 T+P+ (60.6%)*vs.* 41/104 T-P- (39.4%)]. Co-expression was negatively related to early clinical stage (i.e., stages 0 and I; *P* = 0.039). T+P+ breast cancer patients also had worse DFS (95% CI = 1.217–7.499, *P* = 0.017) and OS (*95% CI* = 1.009–9.272, *P* = 0.048). Elevated Twist1 and mutant p53 expression predicted shorter RFS in basal-like patients. Univariate and multivariate analysis identified three variables (i.e., lymph node involvement, larger tumor, and T+P+) as independent prognostic factors for DFS. Lymph node involvement and T+P+ were also independent factors for OS in this cohort. The total risk scores and nomograms were reliable for predicting DFS and OS in breast cancer patients.

**Conclusions:**

Our results revealed that co-expression of mutant p53 and Twist1 was associated with advanced clinical stage, triple negative breast cancer (TNBC) subtype, distant metastasis, and shorter DFS and OS in breast cancer patients. Furthermore, lymph nodes status and co-expression of Twist1 and mutant p53 were classified as independent factors for DFS and OS in this cohort. Co-evaluation of mutant p53 and Twist1 might be an appropriate tool for predicting breast cancer patient outcome.

## Introduction

Breast cancer (BC) is a heterogeneous disease that is classified into different subtypes depending on the expression of the estrogen and progesterone receptors and the amplification of human epidermal growth factor receptor 2 (HER2/ERBB2) (1). In particular, molecular profiling has divided BC into five subtypes: luminal A, luminal B, HER2+, and basal-like and triple-negative breast cancer (TNBC) (2). There are no effective targeted drugs for TNBC due to the lack of estrogen receptor (ERα) or HER2 overexpression. TNBC is one of the most heterogeneous breast cancer subtypes, accounting for 15–20% of all breast cancer. It has poor clinical and pathological features and is considered more aggressive than other BC subtypes, such as luminal A and luminal B (3). Therefore, it is important to identify the molecules and signaling pathways related to TNBC progression. Recent studies demonstrated that the epithelial-to-mesenchymal transition (EMT) process promotes tumor invasion and metastasis in different solid tumor types (4–6). Thus, it is important to identify the transcription factors involved in EMT of TNBC progression (7). EMT is a process characterized by the loss of cell-to-cell adhesion and tight cell junctions, and increased motility and invasion. Twist1 is a major regulator of EMT in breast cancer (8). We previously revealed that Twist1 was overexpressed in breast cancers with higher node status and clinical stage and positively associated with EMT in breast cancer (9).

Mutation of the tumor suppressor gene p53 is found in various cancers, including breast, melanoma, bladder, and colon, particularly in tumors resistant to endocrine therapy or radiotherapy (10–13). Altered p53 protein is prevalently associated with TNBC, and loss of p53 function has been linked to the induction of EMT. Recently, several studies demonstrated that p53-regulated EMT is a critical process in cancer metastasis and chemotherapy resistance. Chang et al. (14) showed that p53 regulates EMT by activating the microRNA miR-200c. Kim et al. (15) demonstrated that p53 loss-of-function or mutation promotes EMT by derepressing Snail. Most noteworthy, van Nes et al. (16) found that Twist1 overexpression is correlated with invasive lobular carcinoma, which is consistent with p53 changes in breast cancer and establishes a mechanistic link between Twist1, p53, and tumor progression. However, studies on the relationship between mutant p53 and Twist1 and their combined role in determining the prognosis and survival of breast cancer patients are scarce. In the present study, we investigated the clinical significance and the combined detection value of mutant p53 and Twist1 in breast cancer patients. We report that mutant p53 and Twist1 co-expression could represent a feasible and effective marker for predicting patient prognosis and survival in breast cancer.

## Materials and Methods

### Patients

A total of 408 breast cancer patients treated at the Cancer Hospital of Shantou University Medical College (SUMC) between 2011 and 2013 were enrolled in this study. None of the patients received any treatment before surgery. Clinicopathological data, including age, menopausal status, tumor size, nodal status, TNM stage, histology, and morphology, were collected. All samples were fixed in buffered formalin and embedded in paraffin for immunohistochemistry (IHC). The DFS and the OS of the patients were quantified. This study was approved by the Medical Ethics Committee of the Cancer Hospital of Shantou University Medical College.

### Immunohistochemistry

All samples were fixed in 10% neutral buffered formalin for 8 to 48 h, followed by dehydration with alcohol and xylene. The dehydrated samples were embedded in paraffin. For immunohistochemical staining, 4-μm-thick paraffin-embedded sections were obtained. The sections were treated with 0.3% H_2_O_2_ for 10 min at room temperature. For antigen retrieval, the sections were heated in 0.01 mmol/L sodium citrate (pH 6.0) in a microwave oven. Sections were then incubated with primary antibodies at 4°C overnight in a humid environment. The stained slides were rinsed thrice in 0.1 mmol/L PBS for 2 min and then incubated for 30 min at room temperature with horseradish peroxidase-conjugated goat anti-rabbit or mouse secondary antibody. Visualization of the stained bands was performed with 3’ 3-diaminobenzidine. Finally, the sections were stained for 5 min with 3,3′-diaminobenzidine (DAB; Fuzhou Maixin Biotechnology Development Co., Ltd., China; DAB-0031) and counterstained with hematoxylin for 1 min. The IHC antibodies were as follows: mutant p53 (Cat. No. 0010-2, Fuzhou Maixin Biotech, Fuzhou, China) and Twist1 (Cat. No. ab50581, Abcam, Cambridge, UK). All immunostained sections were evaluated in a coded manner by the principal author, who was blinded to the patients’ clinicopathologic data. Twist1 expression was detected mainly in the cell membrane and cytoplasm. The expressions were semi-quantitatively determined according to the percentage of positive cancer cells. Staining intensity was classified as four grades: none (0), weak (1), moderate (2) and strong (3). The percentage of positive cancer cells was classified as 4 grades: 0 (0%), 1 (1%-10%), 2 (11%-49%) and 3 (50%-100%). The total score was a product of two scores and the final score of one sample was the mean of 10 microscopic fields. The median score was determined, according to which cancers were categorized into Negative- (score 0-3) and Positive-expression (score 4-6) cancers (9).Brown stained cells in the nucleus were defined as mutant p53 positive cells and the specimens were divided into the following categories: Mutant p53-negative (-), no brown stained; weakly positive (+), <30% cells were mutant p53-positive; moderately positive (++), 30-70% cells were mutant p53-positive; and strongly positive (+++), >70% cells were mutant p53-positive. “-” and “+” were defined as Mutant p53-negative, “++” and “+++” were defined as Mutant p53-positive (17). The following criteria are the definition of molecular typing of breast cancer:TNBC: ER-/PR-/HER2-;HER-2 enriched: ER-/PR-/HER2+;Luminal A: ER+ and/or PR+, HER2-, Ki-67 low expression(≤ 14%);Luminal B: ER+ and/or PR+, HER2-, Ki-67 high expression (>14%) (18, 19).

### Kaplan-Meier Analysis

The free online clinical database, Kaplan-Meier Plotter (http://kmplot.com), was used to analyze the correlation between Twist1 co-expression with wild-type or mutant p53 and prognosis for recurrence-free survival in patients suffering from breast cancer.

### Statistical Analysis

All data were analyzed using SPSS 13.0 statistical software (SPSS Inc., Chicago, IL). The counting data are expressed as the rate using Pearson’s χ^2^ test. Spearman was used for correlation analysis. Survival curves were assessed using the Kaplan-Meier method, and the data were compared using the log-rank test. Univariate and multivariate analyses were used to determine the impact of the variables on patient survival. Two-sided P-values < 0.05 were considered statistically significant.

## Results

### Intersection of Mutant p53 and Twist1 in Clinical and Pathological Features of Breast Cancer

**Figure 1:**
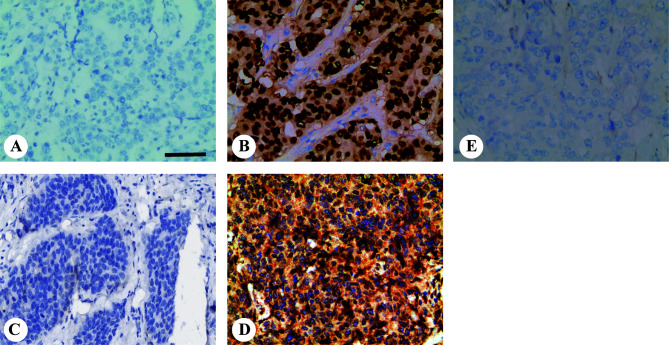
Representative images of mutant p53 and Twist1 immunohistochemical staining in invasive breast carcinomas. Representative immunohistochemistry images for **(A)** mutant p53-negative and; **(B)** mutant p53-positive; **(C)** Twist1-negative and; **(D)** Twist1-positive; **(E)** Negative control IgG staining. Original magnification, 200× The scale bar represents 200 μm.

We stained 408 breast cancer samples to determine the expression of mutant p53 and Twist1 (**Figures 1A–D**) and IgG was used as negative control (**Figure 1E**). Mutant p53 expression was positively associated with invasive ductal carcinoma (IDC) (213/355, 60%, *P* = 0.043). A significant positive relationship was observed between mutant p53 expression and HER2 overexpression (81/119, 68.1%, *P* = 0.009), the luminal B subtype (103/180, 57.2%) and followed by TNBC (36/63, 57.1%) (*P* = 0.004). There were no significant differences with age, menopausal status, lymph node status, TNM stage, histological grade, or ER or PR status. Of the samples analyzed, 220/408 (53.9%) were Twist1-positive. The Twist1-positive rate was significantly higher in ER- (113/147; 76.9%) than in ER+ (107/261; 41.0%) breast cancer (*P* < 0.001). Similarly, it was higher in PR- (127/180; 70.6%) than in PR+ (93/228; 40.8%) breast cancer (*P* < 0.001). Thus, Twist1 was negatively correlated with ER and PR expression. Twist1 expression was significantly higher in TNBC (55/63, 87.3%), follow by HER2-enriched (43/119, 63.9%) and the luminal B (79/180, 43.9%) and luminal A (10/46, 21.7%) subtypes (*P* < 0.001). In contrast, no significant differences were seen with lymph nodal status, TNM stage, histological type, grade, age, or menopausal status. However, Twist1 was positively correlated with mutant p53, E-cadherin, and Ki67 (*P* < 0.001). The relationships between Twist1, mutant p53, E-cadherin, and other biomarkers are shown in **Tables 1** and **2**.

**Table 1:** Association between mutant p53 and Twist1 expression and clinicopathological characteristics in breast cancer patients (n = 408).

Features	P53 (%)		*P*	Twist1 (%)		*P*
	Negative	Positive		Negative	Positive
**Age**						
≤ 60	133 (40.5)	195(59.5)	0.259	154 (47.0)	174(53.0)	0.474
> 60	38 (47.5)	42 (52.5)		34(42.5)	46(57.5)	
**Menopausal status**						
Pre	89 (41.4)	126(58.6)	0.823	96(44.7)	119(55.3)	0.542
Post	82 (42.5)	111(57.5)		92 (47.7)	101(52.3)	
**Tumor size**						
Tis-T1	52 (45.2)	63 (54.8)	0.483	63 (54.8)	52 (45.2)	0.073
T2-T3	102(39.7)	155(60.3)		108 (42.0)	149 (58)	
T4	17 (47.2)	19 (52.8)		17 (47.2)	19 (52.8)	
**Nodal status**						
N0	88 (45.1)	107(54.9)	0.646	99(50.8)	96 (49.2)	0.294
N1	37 (38.9)	58 (61.1)		42(44.2)	53 (55.8)	
N2	28 (40.0)	42 (60.0)		28 (40.0)	42 (60.0)	
N3	18 (37.5)	30 (62.5)		19(39.6)	29 (60.4)	
**Histological grade**						
I	23 (36.5)	40 (63.5)	0.426	31 (49.2)	32 (50.8)	0.285
II	72 (45.6)	86 (54.4)		79 (50.0)	79 (50.0)	
III	76 (40.9)	110(59.1)		78 (41.9)	108(58.1)	
**Histological type**						
IDC	142(40.0)	213(60.0)	**0.043**	161 (45.4)	194(54.6)	0.446
Non IDC	29 (54.7)	24 (45.3)		27 (50.9)	26(49.1)	
**ER**						
Negative	57 (38.8)	90 (61.2)	0.335	34 (23.1)	113(76.9)	**<0.001**
Positive	114(43.7)	147(56.3)		154 (59.0)	107(41.0)	
**PR**						
Negative	77 (42.8)	103(57.2)	0.753	53 (29.4)	127(70.6)	**<0.001**
Positive	94 (41.2)	134(58.8)		135 (59.2)	93 (40.8)	
**HER2**						
Negative	133(46.0)	156(54.0)	**0.009**	145 (50.2)	144(49.8)	**0.010**
Positive	38 (31.9)	81 (68.1)		43 (36.1)	76 (63.9)	
**Molecular subtypes**						
Luminal A	29 (63.0)	17 (37.0)	**0.004**	36 (78.3)	10(21.7)	**<0.001**
Luminal B	77 (42.8)	103(57.2)		101(56.1)	79 (43.9)	
HER2-enriched	38 (31.9)	81 (68.1)		43 (36.1)	76 (63.9)	
TNBC	27 (42.9)	36 (57.1)		8 (12.7)	55 (87.3)	

Statistically significant values (P < 0.05) are in bold.

**Table 2:** Association between Twist1 expression and other molecules in breast cancer patients.

Biomarker	Twist1 (%)		χ^2^	*P*
	Negative (n = 188)	Positive (n = 220)		
**P53**			
Negative	99(57.9)	72(42.1)	16.543	**<0.001**
Positive	89(37.6)	148(62.4)		
**Ki67**				
≤ 14%	45(78.9)	12(21.1)	28.810	**<0.001**
> 14%	143(40.7)	208(59.3)		
**E-cadherin**				
Negative	9(11.2)	71(88.8)	48.583	**<0.001**
Positive	179(54.6)	149(45.4)		.

Statistically significant values (P < 0.05) are in bold.

### Combined Analysis of the Clinical and Pathological Features of Breast Cancer With Mutant p53 and Twist1

Because mutant p53 expression was positively related to Twist1, we classified the study patients into four groups: Twist1- and mutant p53- (T-P-), Twist1+ and mutant p53- (T+P-), Twist1- and mutant p53+ (T-P+), and Twist1+ and mutant p53+ (T+P+). Of the 408 patients, 148 patients belonged to the T+P+ group, while 99 patients were assigned to the T-P- group. It is of note that 37.5% of stage 0 patients (3/8), 45.7% of stage I patients (21/46), 60.6% of stage II patients (63/104), and 68.5% of stage III patients (61/89) were classified as T+P+, indicating that the T+P+ frequency was significantly higher in advanced clinical stages (*P* = 0.039, **Table 3**).

**Table 3:** Correlations between mutant p53 and Twist1 expression and clinicopathologic parameters.

Features	Case (%)		χ^2^	*P*	Case (%)		χ^2^	*P*
	T-P-	T+P+			T+P-	T-P+		
	(n = 99)	(n = 148)			(n = 72)	(n = 89)		
**Age**								
≤60	79(39.7)	120(60.3)	0.062	0.803	54(41.9)	75(58.1)	2.148	0.143
>60	20(41.7)	28(58.3)			18(56.3)	14(43.8)		
**Menopausal status**								
Pre	50(38.5)	80(61.5)	0.300	0.584	39(45.9)	46(54.1)	0.098	0.754
Post	49(41.9)	68(58.1)			33(43.4)	43(56.6)		
**Tumor size**								
Tis-T1	34 (50.0)	34(50.0)	4.552	0.103	18(38.3)	29(61.7)	1.160	0.560
T2-T3	56(35.2)	103(64.8)			46(46.9)	52(53.1)		
T4	9(45.0)	11(55.0)			8(50.0)	8(50.0)		
**Nodal status**								
N0	54(46.6)	62(53.4)	4.199	0.241	34(43.0)	45(57.0)	0.504	0.918
N1	22(36.7)	38(63.3)			15(42.9)	20(57.1)		
N2	15(34.1)	29(65.9)			13(50.0)	13(50.0)		
N3	8(29.6)	19(70.4)			10(47.6)	11(52.4)		
**Clinical stage**								
0	5(62.5)	3(37.5)	8.345	**0.039**	1(33.3)	2(66.7)	0.344	0.076
I	25(54.3)	21(45.7)			18(46.2)	21(53.8)		
II	41(39.4)	63(60.6)			27(42.9)	36(57.1)		
III	28(31.5)	61(68.5)			26(46.4)	30(53.6)		
**Histological grade**								
I	15(38.5)	24(61.5)	2.308	0.315	8(33.3)	16(66.7)	1.700	0.427
II	43(46.2)	50(53.8)			29(44.6)	36(55.4)		
III	41(36.0)	73(64.0)			35(48.6)	37(51.4)		
**Histological type**								
IDC	17(54.8)	14(45.2)	3.215	0.073	12(54.5)	10(45.5)	0.995	0.319
Non-IDC	82(38.0)	134(62.0)			60(43.2)	79(56.8)		
**ER**								
Negative	19(20.2)	75(79.8)	24.943	**<0.001**	38(71.7)	15(28.3)	23.260	**<0.001**
Positive	80(52.3)	73(47.7)			34(31.5)	74(68.5)		
**PR**								
Negative	30(27.3)	80(72.7)	13.547	**<0.001**	47(67.1)	23(32.9)	25.187	**<0.001**
Positive	69(50.4)	68(49.6)			25(27.5)	66(72.5)		
**HER2**								
Negative	84(46.9)	95(53.1)	12.69	**<0.001**	49(44.5)	61(55.5)	0.004	0.948
Positive	15(22.1)	53(77.9)			23(45.1)	28(54.9)		
**Molecular subtypes**								
Luminal A	24(82.8)	5(17.2)	44.415	**<0.001**	5(29.4)	12(70.6)	27.07	**<0.001**
Luminal B	53(49.1)	55(50.9)			24(33.3)	48(66.7)		
HER2-enriched	15(22.1)	53(77.9)			23(45.1)	28(54.9)		
TNBC	7(16.7)	35(83.3)			20(95.2)	1(4.8)		

Statistically significant values (P < 0.05) are in bold.

A comparison between T+P+ and T-P- groups showed that more ER- patients (75/94, 79.8%) and PR- patients (80/110, 72.7%) were T+P+ (*P* < 0.001). Similarly, significantly higher mutant p53 and Twist1 expression levels were observed with the HER2+ (77.9% T+P+ *vs.* 22.1% T-P-, *P* < 0.001) and TNBC (83.3% T+P+ *vs.* 16.7% T-P-, *P* < 0.001) subtypes.

As shown in **Table 4**, patients with T+P+ had higher Ki67, E-cadherin, and VEGF-C expression than the T-P- group (*P* < 0.001). There were no statistically significant differences between the T+P- and T-P+ groups in terms of age, menopausal status, tumor size or clinical stage.

**Table 4:** Correlations of mutant p53 and Twist1 expression with other molecules in breast cancer patients.

Biomarker	Case (%)		χ^2^	*P*	Case (%)		χ^2^	*P*
	T-P-	T+P+			T+P-	T-P+	
	(n = 72)	(n = 172)			(n = 48)	(n = 116)	
**Ki67**								
≤ 14%	30(83.3)	6(16.7)	32.827	**<0.001**	6(28.6)	15(71.4)	2.548	0.110
> 14%	69(32.7)	142(67.3)			66(47.1)	74(52.9)		
**E-cadherin**								
Negative	4(8.7)	42(91.3)	23.185	**<0.001**	29(85.3)	5(14.7)	28.702	**<0.001**
Positive	95(47.3)	106(52.7)			43(33.9)	84(66.1)		
**VEGF-C**								
Negative	45(57.0)	34(43.0)	13.782	**<0.001**	14(34.1)	27(65.9)	2.488	0.115
Positive	54(32.1)	114(67.9)			58(48.3)	62(51.7)		

Statistically significant values (P < 0.05) are in bold.

### Univariate and Multivariate Survival Analysis for Predicting DFS and OS

**Figure 2:**
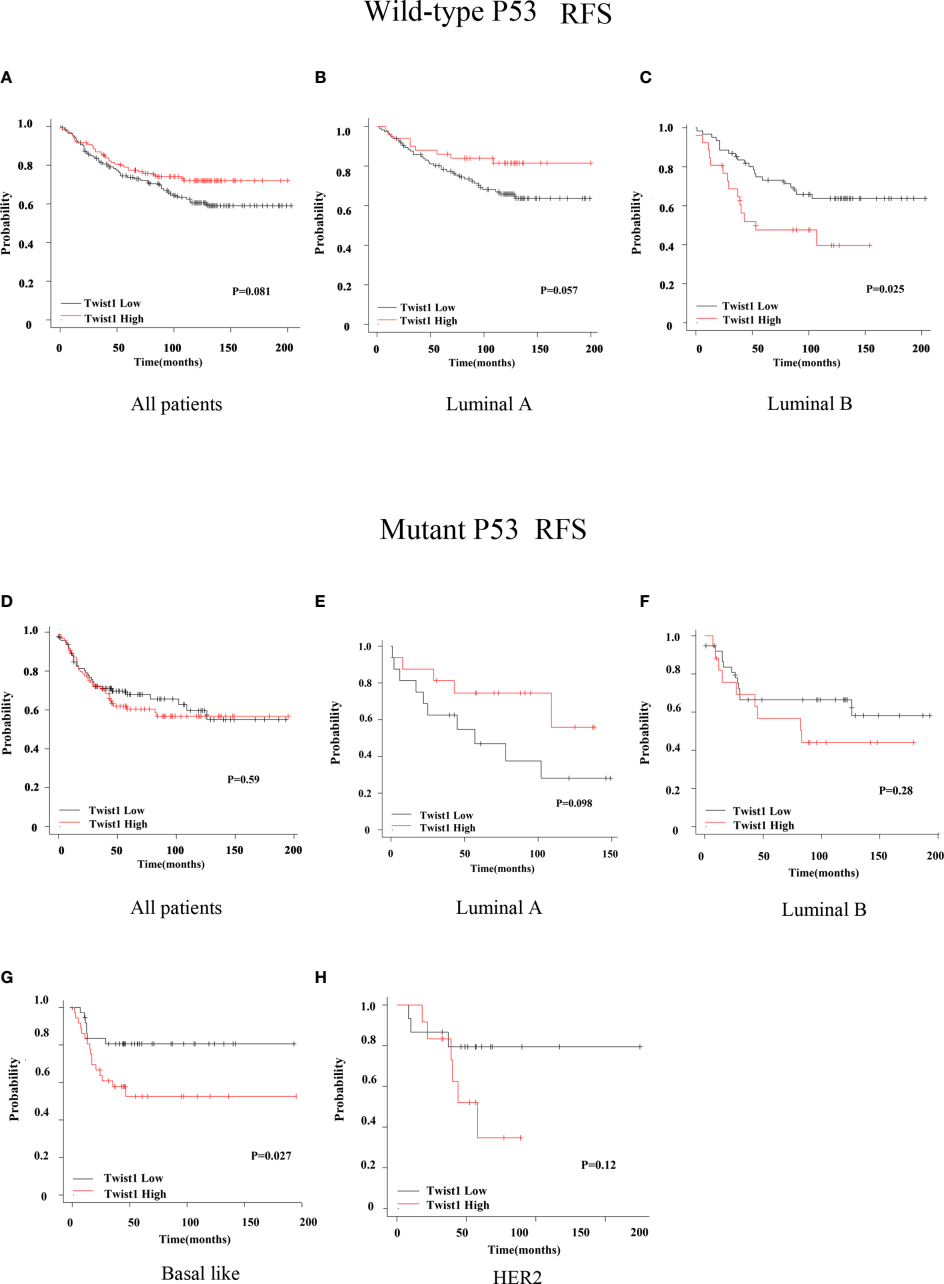
Comparison of RFS for wild-type or mutant p53 breast cancer patients with different Twist1 levels. **(A–C)** Different Twist1 expression levels did not differ in RFS (*P* > 0.05) among all wild-type p53 breast cancer patients **(A)** and luminal A **(B)** and luminal B **(C)** subtypes. **(D–H)** Different Twist1 expression levels did not differ in RFS (*P* > 0.05) among all mutant p53 breast cancer patients **(D)** and luminal A **(E)**, luminal B **(F)**, and HER2 **(H)** subtypes. **(G)** High Twist1 expression had the worst RFS (*P* < 0.05) among the mutant p53, basal-like breast cancer patients.

**Figure 3:**
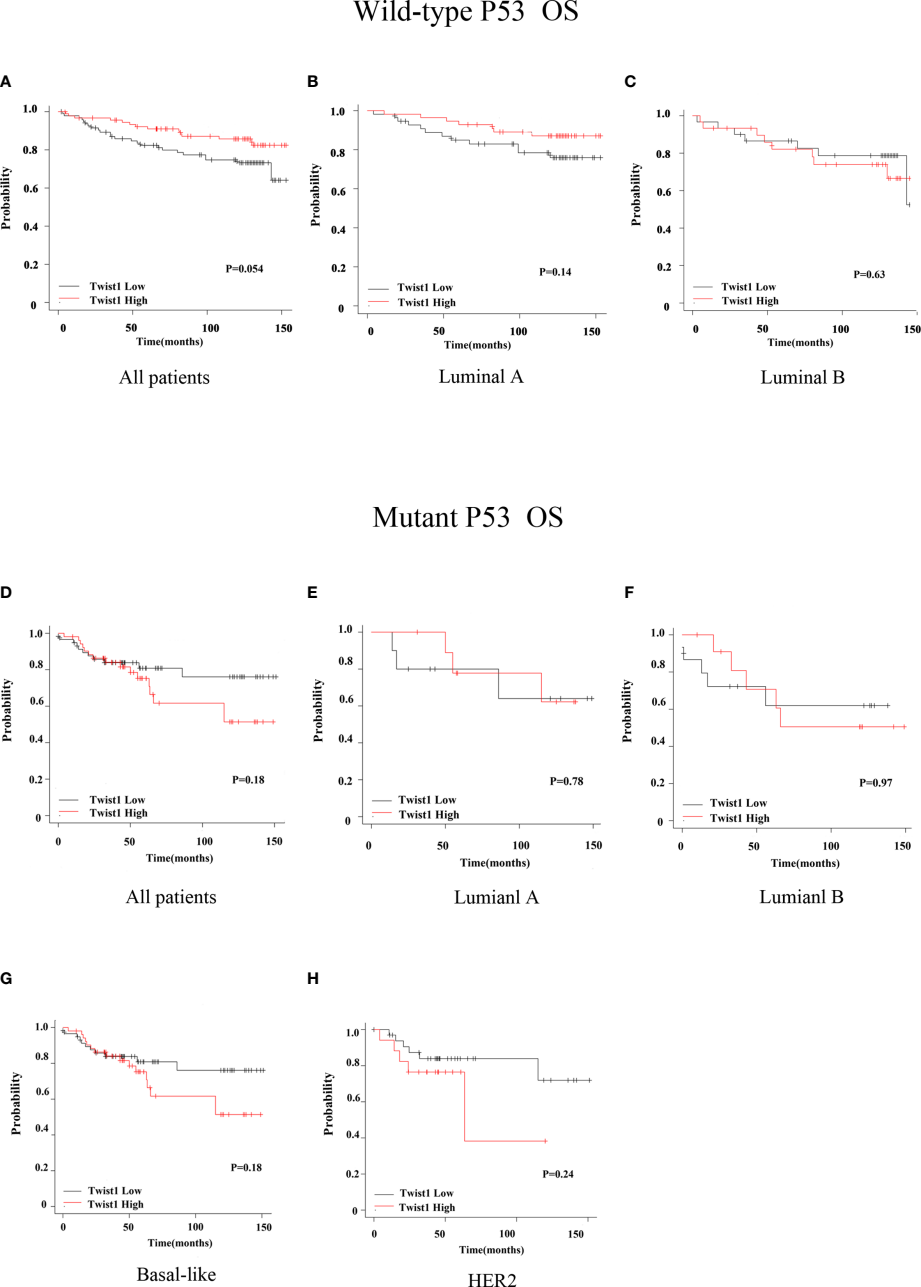
Comparison of OS for wild-type or mutant p53 breast cancer patients with different Twist1 levels. **(A–C)** Different Twist1 expression levels did not differ in OS (*P* > 0.05) among all wild-type p53 breast cancer patients **(A)** and luminal A **(B)** and luminal B **(C)** subtypes. **(D–G)** Different Twist1 expression levels did differ OS (*P* > 0.05) among all mutant p53 breast cancer patients **(D)** and luminal A **(E)**, luminal B **(F)**, and basal-like **(G)** subtypes **(H)** HER2 subtype.

We next assessed the prognostic value of Twist1 with or without p53 mutation in breast cancer using the Kaplan-Meier plotter. The results demonstrated that elevated Twist1 expression combined with mutant p53 predicted shorter RFS for basal-like breast cancer patients (*P* = 0.027) (**Figure 2G** **)**; however, there was no difference for the OS (*P* = 0.18) (**Figure 3G** **)**. There were no statistical differences in RFS and OS for the other groups of patients (**Figures 2** and **3**).

**Figure 4:**
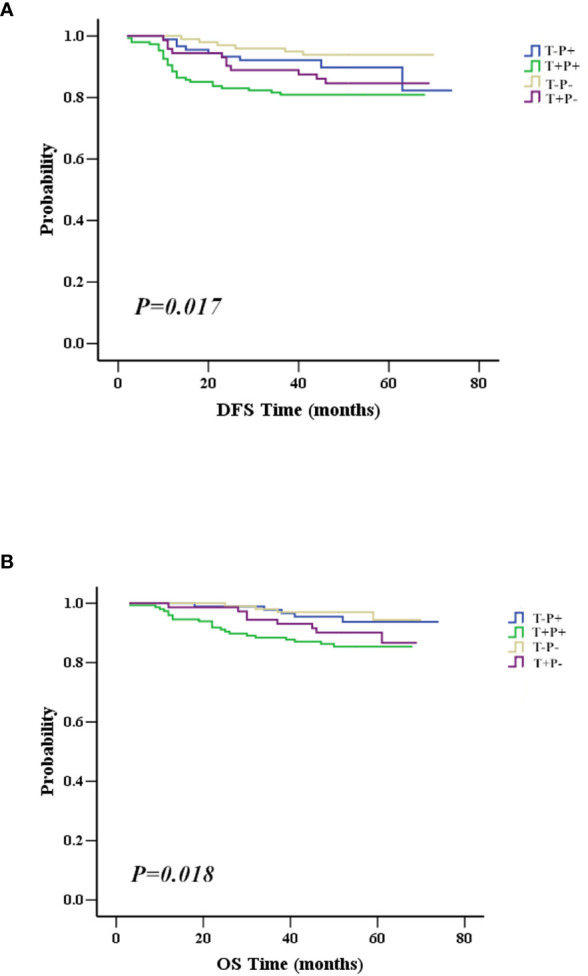
Association between mutant p53 and Twist1 co-expression with survival. **(A)** Patients with mutant p53 and Twist1 co-expression (T+P+) have worst DFS than patients in other groups, including Twist1- and mutant p53- (T-P-), Twist1+ and mutant p53- (T+P-), and Twist1- and mutant p53+ (T-P+) (*P* = 0.017). **(B)** Patients with co-expression of mutant p53 and Twist1 (T+P+) have worst OS compared to patients in other groups, including Twist1- and mutant p53- (T-P-), Twist1+ and mutant p53- (T+P-), and Twist1- and mutant p53+ (T-P+) (*P* = 0.018).

To investigate which factors could predict the clinical outcome of breast cancer patients, we performed univariate and multivariate survival analysis. DFS was followed up for 2 to 74 months and OS for 3 to 74 months. In our cohort, 148 patients belonged to the T+P+ group, while 99 patients were T-P-. The variables included in the analysis were tumor size, node stage, molecular subtype, and the different combinations of Twist1 and mutant p53 (i.e., T-P-, T+P-, T-P+, and T+P+). **Tables 5** and **6** show the main results of the survival analysis. By univariate analysis, we found the node status was positively related with DFS (N1 *vs.* N0, HR = 3.749, 95% CI = 1.476–9.524, *P* = 0.005; N2 *vs.* N0, HR = 7.608, 95% CI = 3.154–18.350, *P* < 0.001; N3 *vs.* N0, HR = 13.977 95% CI = 5.871–33.277, *P* < 0.001). The multivariate analysis demonstrated that node status was an independent factor that influenced DFS (N1 *vs.* N0, HR = 2.757, 95% CI = 1.069–7.112, *P* = 0.036; N2 *vs.* N0, HR = 5.386, 95%CI = 2.134–13.595, *P* < 0.001; N3 *vs.* N0, HR = 10.757, 95% CI = 4.286–27.000, *P* < 0.001). A larger tumor size was another independent risk factor for DFS in breast cancer (**Table 5**). Multivariate analysis demonstrated that node status was also an independent factor influencing OS (N1 *vs.* N0, HR = 8.522 95% CI = 1.789–40.606, *P* = 0.007; N2 *vs.* N0, HR = 13.717, 95% CI = 2.780–67.686, *P* = 0.001; N3 *vs.* N0, HR = 41.619, 95% CI = 8.943–193.679, *P* < 0.001). However, there were no significant differences between tumor size, molecular typing, and OS by multivariate analysis. Patients with co-expression of mutant p53 and Twist1 (T+P+) had worse DFS (*P* = 0.017) and OS (*P* = 0.018) compared to patients in the other groups (i.e., T-P-, T+P-, and T-P+) (**Figures 4A, B** **)**.

**Table 5:** Disease-free survival of patients diagnosed with breast cancer.

Features	Univariate			Multivariate		
	Hazard ratio	95% CI	*P*	Hazard ratio	95% CI	*P*
**Tumor Size**						
T2-T3 *vs.*T0-T1	9.843	2.381,40.697	**0.002**	4.399	1.028, 18.825	**0.046**
T4 *vs.* T0-T1	21.516	4.815,96.151	**<0.001**	7.366	1.547,35.067	**0.012**
**Nodal status**						
N1 *vs.* N0	3.749	1.476, 9.524	**0.005**	2.757	1.069,7.112	**0.036**
N2 *vs.* N0	7.608	3.154,18.350	**<0.001**	5.386	2.134,13.595	**<0.001**
N3 *vs.* N0	13.977	5.871,33.277	**<0.001**	10.757	4.286,27.000	**<0.001**
**Molecular subtype**						
Luminal B *vs.* luminal A	1.840	0.549, 6.172	0.323	1.107	0.318,3.856	0.873
Her2 *vs.* Luminal A	2.791	0.829, 9.396	0.097	1.940	0.548,6.869	0.304
TNBC *vs.* Luminal A	2.899	0.808,10.394	0.102	2.312	0.604,8.852	0.221
**Case**						
T+P- *vs.* T-P-	2.612	0.966,7.062	0.059	1.724	0.627,4.739	0.291
T-P+ *vs.* T-P-	1.907	0.693,5.249	0.211	1.696	0.613,4.687	0.309
T+P+ *vs.* T-P-	3.577	1.480,8.648	**0.005**	3.020	1.217,7.499	**0.017**

Statistically significant values (P < 0.05) are in bold.

**Table 6:** Overall survival of patients diagnosed with breast cancer.

Features	Univariate			Multivariate		
	Hazard ratio	95% CI	P	Hazard ratio	95% CI	P
**Tumor Size**						
T2-T3 *vs.* T0-T1	6.354	1.511, 26.720	**0.012**	1.868	0.422, 8.273	0.410
T4 *vs.* T0-T1	14.961	3.232,69.259	**0.001**	3.078	0.606,15.637	0.175
**Node status**						
N1 *vs.* N0	9.930	2.145,45.971	**0.003**	8.522	1.789,40.606	**0.007**
N2 *vs.* N0	15.012	3.289,68.524	**<0.001**	13.717	2.780,67.686	**0.001**
N3 *vs.* N0	40.318	9.308,174.632	**<0.001**	41.619	8.943,193.679	**<0.001**
**molecular subtype**						
luminal B *vs.* luminal A	1.571	0.352,7.022	0.554	0.825	0.174,3.917	0.809
Her2 *vs.* luminal A	2.839	0.645,12.494	0.168	1.965	0.416,9.281	0.394
TNBC *vs.* luminal A	3.915	0.858,17.876	0.078	3.065	0.612,15.344	0.173
**Case**						
T+P- *vs.* T-P-	2.779	0.837,9.228	0.095	1.694	0.500,5.742	0.398
T-P+ *vs.* T-P-	1.404	0.377,5.228	0.613	1.194	0.319,4.473	0.793
T+P+ *vs.* T-P-	3.914	1.343,11.412	**0.012**	3.059	1.009,9.272	**0.048**

Statistically significant values (P < 0.05) are in bold.

**Figure 5:**
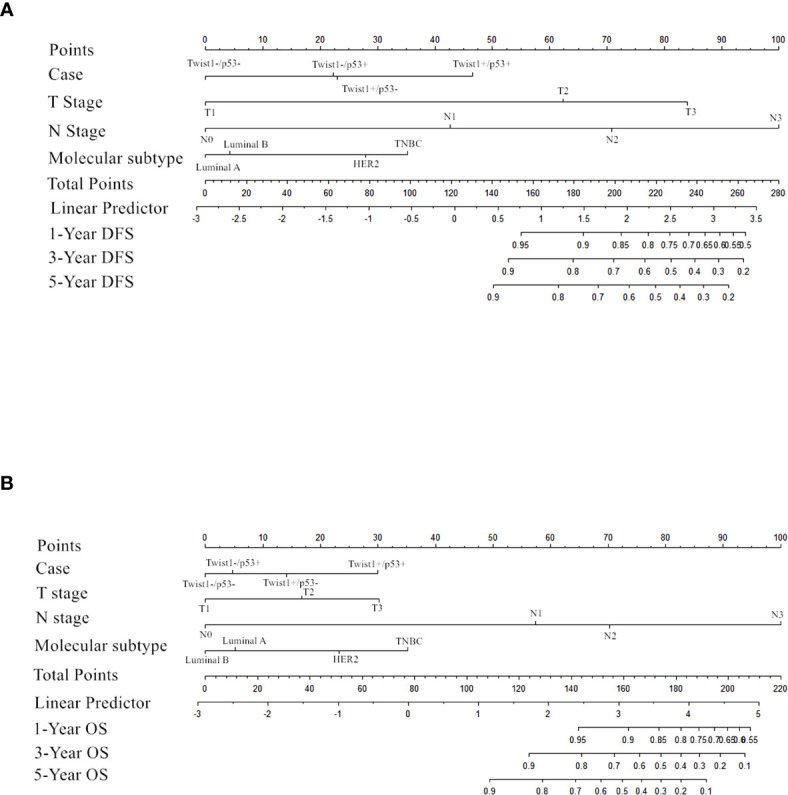
Nomogram for predicting DFS **(A)** and OS **(B)** in the cohort.

Importantly, the multivariate analysis demonstrated that the T+P+ group had a worse DFS (*95% CI* = 1.217–7.499, *P* = 0.017) and OS (*95% CI* = 1.009–9.272, *P* = 0.048) than the T-P-group, but the T+P- and T-P+ groups were not statistically different from the T-P- group. Univariate and multivariate analysis identified three variables (high node stage, larger tumor size, and Twist1 and mutant p53 co-expression) as independent risk factors for DFS and node involvement and T+P+ as independent risk factors for OS in breast cancer. Therefore, total risk scores and nomograms were considered suitable for predicting the DFS and OS of breast cancer patients (**Figure 5**).

## Discussion

Twist1 is a master transcription factor that induces migration and invasion and promotes EMT in various cancer cells (20–22). Wild-type p53 is a transcription factor that promotes cell cycle arrest, DNA repair, cellular senescence, and apoptosis (23). p53 inhibits cancer cell proliferation and metastasis; however, mutant TP53 has a transforming function. Previous studies have demonstrated that mutant p53 is overexpressed and accumulates to high levels in cancer cells, and is highly correlated with EMT (24). Moreover, Twist1 overexpression can inhibit oncogene-induced premature senescence by blocking key regulators of the p53 and Rb-dependent pathways (25). Numerous studies have shown that there is a regulatory relationship between Twist1 and p53. For instance, Shiota et al. (26) suggested that Twist1 suppresses the DNA-binding activity of p53. Moreover, Kogan-Sakin et al. (27) revealed that Twist1 might be upregulated following p53 mutation in cancer cells. However, there are no reports on the combined detection of Twist1 and mutant p53 at the protein level and its prognostic impact on breast cancer patients.

We analyzed the expression patterns of mutant p53 and Twist1 in breast cancer to explore the role of these molecular markers in breast carcinogenesis, their influence on prognosis, and clinical practicability in breast cancer. Our study showed that mutant p53 expression was positively associated with invasive ductal carcinoma (IDC). Mutant p53 was highest in the HER2+ breast cancer subtype, followed by the luminal B and TNBC subtypes. Twist1 positivity was significantly higher in TNBC patients. Consistent with previous studies (9, 28), we further confirmed that high Twist1expression was associated with larger tumor size, higher node involvement, and shorter DFS. Most importantly, we found, for the first time, that Twist1 was positively correlated with mutant p53, E-cadherin, and Ki67. The association of mutant p53 with Twist1 in clinical tissues was supported by the work of Yang-Hartwich et al. (29), who showed that wild-type p53 promoted Twist1 protein degradation and inhibited EMT, maintaining the epithelial phenotype. Interestingly, a preclinical study by Li et al. (30) found that Twist1 had noteworthy impacts on p53 and p21 induction, which were similar to our current results. Maestro et al. (31) found that Twist regulated p53 indirectly by modulating the ARF/MDM2/p53 pathway. Similarly, Piccinin et al. (32) found that Twist1 binds to the p53 C terminus through the Twist box to inactivate p53 in mesenchymal tumors. Conversely, Twist promotes the reprogramming of glucose metabolism in MCF10A-Twist cells and Twist-positive breast cancer cells by inhibiting the p53 pathway (33). Taken together, these findings suggest that a mutual regulation mechanism might exist between mutant p53 and Twist1. However, the clinical significance of co-expression of two proteins has not been clearly defined.

In this study, we also investigated the relationship and clinical significance of mutant p53 and Twist1. Our results suggested that co-overexpression of mutant p53 and Twist1 in breast cancer was significantly associated with larger tumor size, greater lymph node involvement, and more advanced TNM stage. Co-expression of mutant p53 and Twist1 (T+P+ group) was significantly higher in TNBC, followed by HER2-enriched, suggesting the aggressiveness of T+P+ tumors. Previously, Twist 1 overexpression was shown to be associated with poor prognosis in breast cancer (20, 34). Similarly, abnormal p53 expression has been widely accepted as a poor prognostic factor in breast cancer (35). Moreover, Sanambar et al. (36) found that p53 mutation detected by immunohistochemistry could effectively predict the prognosis of breast cancer patients. In our study, regression analysis was used to assess the role of mutant p53 by immunohistochemistry. As expected, the patients in the T+P+ group presented the worst DFS and OS by univariate analysis. Furthermore, our results demonstrated that co-expression of Twist1 and mutant p53 (T+P+) is an independent prognostic factor for both DFS and OS in breast cancer patients using multivariate analysis. Similarly, Kaplan-Meier analysis using the Kaplan-Meier plotter database demonstrated that co-expression of Twist1 and mutant p53 predicted shorter RFS in patients with basal-like breast cancer. However, due to the small sample size of the database, we did not find a statistical difference between Twist1 with mutant or wild-type p53 and RFS and OS in other subtypes.

A nomograph was established based on the survival analysis, and risk scores for DFS and OS time were calculated according to the Cox regression coefficient. Internal validation patients were randomly selected from the total population, and the verification confirmed that the risk score was related to DFS and OS time, showing the nomogram’s reliability. However, the weakness is the lack of validation queues in our research. Future research should enroll more cases to further verify the nomogram.

This study is the first report showing that co-expression of Twist1 and mutant p53 could be used to evaluate treatment efficacy and prognosis in breast cancer patients. The relationship between the two proteins needs further study, such as the relationship between the specific mutation site of the p53 gene and breast cancer and the interactive regulatory mechanism of mutant p53 and Twist1. Using small molecular inhibitors that inhibit the expression of Twist1 and mutant p53 may be one strategy for targeted treatment of TNBC (37).

## Data Availability Statement

The raw data supporting the conclusions of this article will be made available by the authors, without undue reservation.

## Ethics Statement

The studies involving human participants were reviewed and approved by Medical Ethics Committee of the Cancer Hospital of Shantou University Medical College. Written informed consent for participation was not required for this study in accordance with the national legislation and the institutional requirements.

## Author Contributions

Conception and design: G-JZ and X-LW. Administrative support: G-JZ. Provision of study materials or patients: G-JZ. Collection and assembly of data: Y-QZ. Data analysis and interpretation: Y-QZ and FZ. All authors contributed to the article and approved the submitted version.

## Funding

This work was partly supported by the National Natural Science Foundation of China (No. 81602345 and No. 91859120), the Science and Technology Planning Project of Guangdong Province (No. 2016A020215145), the Science and Technology Planning Project of Shantou (No. 190917085269842), the Start-Up Funds from Xiamen University, the Science and Technology Project of Xiamen Municipal Bureau of Science and Technology (No.3502Z20199047), the Natural Science Foundation of Fujian Province of China (No. 2020J01015), Fujian Major Scientific and Technological Special Project for “Social Development” (No.2020YZ016002).

## Conflict of Interest

The authors declare that the research was conducted in the absence of any commercial or financial relationships that could be construed as a potential conflict of interest.
